# Last Call for Replacement of Antimicrobials in Animal Production: Modern Challenges, Opportunities, and Potential Solutions

**DOI:** 10.3390/antibiotics9120883

**Published:** 2020-12-09

**Authors:** Aneta Nowakiewicz, Przemysław Zięba, Sebastian Gnat, Łukasz Matuszewski

**Affiliations:** 1 Sub-Department of Veterinary Microbiology, Institute of Preclinical Veterinary Sciences, Faculty of Veterinary Medicine, University of Life Sciences, Akademicka 12, 20-033 Lublin, Poland; sebastian.gnat@up.lublin.pl; 2 State Veterinary Laboratory, Droga Męczenników Majdanka 50, 20-325 Lublin, Poland; przemekzieba@gmail.com; 3 Department of Pediatric Orthopedics and Rehabilitation, Faculty of Medicine, Medical University, Gębali 6, 20-093 Lublin, Poland; lukasz.matuszewski@umlub.pl

**Keywords:** antimicrobial resistance, bacteriophages, antimicrobial peptides, animal production

## Abstract

The constant market demand for easily available and cheap food of animal origin necessitates an increasing use of antibiotics in animal production. The alarming data provided by organizations monitoring drug resistance in indicator and pathogenic bacteria isolated from humans and animals indicate a possible risk of a return to the preantibiotic era. For this reason, it seems that both preventive and therapeutic measures, taken as an alternative to antimicrobials, seem not only advisable but also necessary. Nevertheless, the results of various studies and market analyses, as well as difficulties in the implementation of alternative substances into veterinary medicine, do not guarantee that the selected alternatives will completely replace antimicrobials in veterinary medicine and animal production on a global scale. This publication is a brief overview of the drug resistance phenomenon and its determinants, the steps taken to solve the problem, including the introduction of alternatives to antimicrobials, and the evaluation of some factors influencing the potential implementation of alternatives in animal production. The review also presents two groups of alternatives, which, given their mechanism of action and spectrum, are most comparable to the effectiveness of antibiotics, as emphasized by the authors.

## 1. Introduction

**Figure 1:**
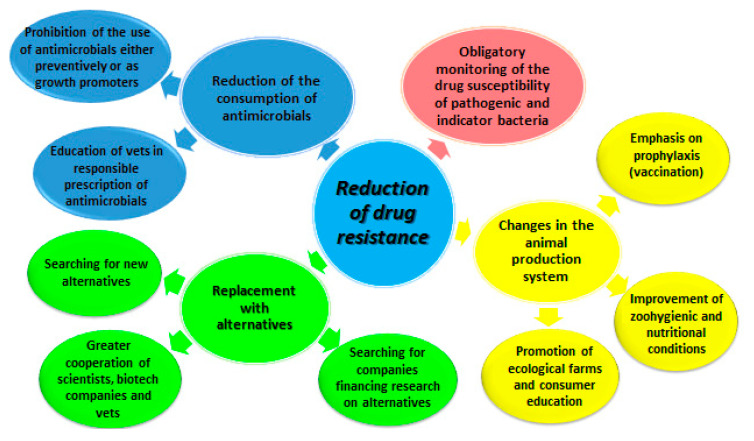
Actions for reduction of microbial resistance to drugs (based on available reports, literature, and the RONAFA (European agencies) opinion.

For over 80 years, humans have had a weapon to fight the microbes responsible for infectious diseases that have devastated the world’s populations for centuries. The discovery of penicillin started a completely new era of availability of new antibacterial substances [1]. The following years brought new solutions related to either the discovery of new substances or the production of derivatives of existing agents. Thus, the application of antimicrobials increased significantly [2]. In addition to the treatment of infectious diseases in humans and animals, there was another positive effect of the application of antimicrobials [3]. They started to be used as growth promoters in animal production (GPs), which resulted in a significant increase in the application of this type of product and prompted the market to search for other easily accessible novel substances [3]. Despite the side effects associated with the use of subtherapeutic doses, observed as early as in the 1940s, no appropriate procedures to eliminate the effects of rapidly emerging resistance were developed before the 1980s [4]. In response to the severity of the problem, the World Health Organization (WHO) identified antibiotic resistance as one of the global threats to public health. The “No time to wait” report, published in 2019 [5], estimated that if no action is taken, 10 million people will die annually from multidrug-resistant microbial infections by 2050. The greatest risk, according to the report, is the resistance to the so-called “last resort antimicrobials”, which are the last line of defense against common diseases [6]. One of the main causes of the emerging drug resistance is the unskillful and often excessive and unnecessary use of antimicrobials in animal production [7]. Therefore, many global activities that are focused on the assessment of drug resistance, as well as programs for the reduction of the use of antibiotics, have been undertaken over the last two decades, primarily in animal production (Figure 1, Table 1). One of the essential elements of these activities consists of the replacement of antimicrobials with alternative treatments [8]. However, although various substances, e.g., probiotics and prebiotics, have long been subjected to analyses, yielding preparations with specific molecular particles that stimulate the immune system (e.g., CpGs) [9], no satisfactory compromise has been reached between this group of preparations and antibiotics, in particular in terms of their effectiveness, efficiency, economic balance, and public satisfaction [10]. Therefore, the aim of this review is to highlight the rationale behind the necessary reduction of the use of antimicrobials and developing, testing, and implementing new alternatives to antibiotics that are currently used in animal production. The difficulties associated with this process are emphasized as well. Since a large group of alternatives is currently being tested and used in both human medicine and animal production, we will focus only on two groups targeted at microorganisms, which we believe have the most similar mode of action to antibiotics. We will also try to present their advantages and disadvantages.

## 2. Antimicrobials and Antibiotics: Why Did Antimicrobial Resistance Appear?

Antimicrobials in agriculture were first used upon the introduction of synthetic sulfonamides to the market before the outbreak of the Second World War [11]. Antibiotics were then used not only therapeutically and in animal husbandry but also in plant sprays and for the prevention of food spoilage [12,13]. Initially, the USA became a leader in the production and nontherapeutic use of antibiotics. However, already in the 1950s, antibiotics were used as GPs, mainly in poultry farming in some European countries [3]. The high density of animals kept in a small space resulted in numerous falls and losses. Then, the administration of antibiotics to healthy animals not only reduced this negative phenomenon but also accelerated animal growth and increased feed conversion [3,14,15]. In the other livestock production sectors, the use of antibiotics as GPs was approved with less enthusiasm by European farmers [4]. However, a market analysis conducted in the late 1950s in a few European countries showed that more than 50% of farmed pigs, poultry, and calves were administered antibiotics as growth promoters [16].

On the other hand, the use of antibiotic GPs had a measurable positive effect, i.e., a significant reduction of meat prices [17]. Therefore, the use of antimicrobials in animal production continued to increase both in the USA and in European countries [4,17] since the postwar market required fast and efficient food production [7,18].

Initially, only the positive aspect of the use of antibiotics was highlighted. It was only in the late 1950s that the negative effect of the permanent use of antibiotics started to be discernible [19]. Originally, the interest was focused on antibiotic residues in food only, due to the dissatisfaction of consumers, who perceived the negative toxic or carcinogenic effects of all drug residues. In response to the alarming results of analyses of antibiotic residues in milk and meat, the use of tetracycline as a food preservative was banned in Germany [20]

Although the first penicillin-resistant *Staphylococcus aureus* (penicillinase-producing strains) appeared only a few years after the discovery of this antibiotic, the possibility of horizontal gene transfer (HTG) between bacteria was first discussed in the late 1950s [21,22]. The HTG phenomenon, increasing the variability of bacteria, facilitates bacterial survival through the rapid acquisition of genes of antibiotic resistance mechanisms [21,22]. Despite the emergence of a significant number of reports on the increasing resistance of bacteria and the likelihood of return of human and animal medicine to the preantibiotic era [14,19,23], no major action was taken on a wider scale and there was no global scheme of handling this issue at the end of the 1970s. The unjustified use of antibiotics was additionally supported by the black market and gaps in the regulations on prescribing antimicrobials [4,14]. Unfortunately, the circulation of unregistered drugs and the use of antibiotics without prescription still exist today, constantly increasing the problem of resistance [24].

## 3. Yesterday and Today: Activities for Elimination of the Effects of Excessive Antibiotic Therapy

The policy of limitation of the use of antibiotics as growth promoters was introduced only in the 1980s. At the beginning, the change was only associated with the trend of consumers’ return to safe and healthy food, especially in highly developed countries [25]. In Eastern Europe, the problem was purely academic for a long time. The political emphasis on the intensification of livestock production, related to the economic race on both sides of the Iron Curtain, only exacerbated this phenomenon [4]. The increased drug resistance began to be perceived as a global threat only after the changes in the political and economic relationships between European countries in the 1990s and the successive incorporation of individual countries into the European Union [26]. The first efficient actions were taken by the Scandinavian countries, which imposed a partial or complete ban on the use of antibiotics as growth promoters, introduced alternative methods for changes in animal husbandry conditions (Sweden) [27], or increased the intensification of vaccination (Norway) [28]. In Denmark, an increase in cross-resistance to vancomycin, resulting from resistance to avoparcin, was observed for the first time [29]. It resulted in a ban on the use of this substance in animals as early as 1995 [30]. Finally, on 1 January 2006, the use of the last four antibiotics as feed additives (monensin, salinomycin, avilamycin, flavophospholipol) was prohibited in EU countries [3]. Nowadays, besides the EU parliament, at least three European agencies, i.e., the European Center for Disease Prevention and Control (ECDC), the European Food Safety Authority (EFSA), and the European Medicines Agency (EMA), control the consumption of antimicrobials, the level and development of resistance, and implementation of stewardship programs for the education of antimicrobial resistance in EU countries. Since 2004, annual reports on the level of resistance of zoonotic and indicator bacteria, collected and evaluated according to a harmonized protocol for EU countries, have been delivered.

Moreover, in April 2010, EMA started the project “The European Surveillance of Veterinary Antimicrobial Consumption (ESVAC)”, collecting data on antimicrobials used in animals in EU countries. These data are used for the assessment of the risk associated with the trends and type of resistance spreading in EU countries. Besides the annual comparative analysis, the data focus primarily on early detection of resistance to last-line antimicrobials and registration of bacteria with resistance phenotypes recognized by WHO as priority pathogens [31]. For example, the DANMAP program implemented in Denmark (Table 1) demonstrated a significant decrease in vancomycin-resistant *Enterococcus* indicator strains isolated from animals for 11 years since 2006, which was associated with the withdrawal of avoparcin [32,33]. Moreover, a decrease in the consumption of the most important life-saving drugs was recorded in 2011–2017: the use of cephalosporins, polymyxins, and fluoroquinolones declined by 20.9%, 66.4%, and 10.3%, respectively [34]. However, these reports also indicate a growing trend of resistance to some antibiotics in some countries over the past five years [35].

In addition to the pan-European measures, individual countries have undertaken actions to monitor the consumption of antimicrobials in animal production. Some countries, e.g., Belgium, France, UK, Sweden, Denmark, and the Netherlands, have increased their efforts for better control of the prescription and administration of antimicrobials by veterinarians, limitation of the use of antibiotics for humans, especially drugs from the group of critically important antimicrobials (CIA) in veterinary medicine, and coordination of the use of these drugs [36].

In other countries, the problem has not been solved to date. In the US, medically important antibiotics were withdrawn from use as growth promoters in 2017; however, as shown by practice and data on the sale of antibiotics, the border between the use of drugs as growth promoters, prophylaxis, metaphylaxis, and therapy is highly controversial [8,34]. The O’Neill [37] report estimated that only 1/3 of antibiotics are used in livestock for therapeutic purposes, and the remaining part includes prophylaxis (disease prevention) or application as growth promoters [37,38]. This is also the case for BRICS countries (Brazil, Russia, India, China, and South Africa), where animal production is intensified, the sales of antibiotics are increasing at an alarming rate, and their use is expected to increase by 67% or even 99% by 2030 [37].

The predictions and successive monitoring of the resistance of microorganisms, mainly those isolated from farm animals (annual reports from individual countries included in Table 1) [39], prompted WHO and other organizations to develop guidelines indicating the most important points in the elimination of drug resistance. The results of this work were presented in the Interagency Coordination Group (IACG) report on Antimicrobial Resistance, identifying the main targets and predictions for the development of resistance and mortality associated with the treatment of MDR infections [5]. The IACG recommended the acceleration of activities in all countries through the development and implementation of One Health National Antimicrobial Resistance Action Plans and a consistent withdrawal of antibiotic growth promoters, including immediate discontinuation of drugs recognized by WHO as critically important antimicrobials (CIAs) for human medicine [40]. Another important element of the IACG report was the calls on research funders and other stakeholders to contribute to current research and development efforts for new antimicrobials, diagnostics, vaccines, waste management tools, and safe and effective alternatives to antimicrobials. The report also underlined the need to strengthen implementation and operational research, as well as the coordination of investigations and collaboration, in the One Health context [5].

In turn, the European agencies EMA and EFSA published their joint opinion (also known as the RONAFA opinion) [33] that indicated three main elements, i.e., reduction of the use of antimicrobials in animals to the minimum necessary, replacement thereof with such alternatives as vaccines, probiotics, prebiotics, bacteriophages, and organic acids, and/or reconstruction of the livestock system by the implementation of farming practices to prevent the introduction and spread of diseases (Figure 1). The One Health National Antimicrobial Resistance Action Plans against AMR antimicrobial resistance [41] was first adopted in 2017 and imposed the implementation of specific solutions on the European Commission. The program recommended development and innovation by filling the current gaps in knowledge, providing novel solutions and tools to prevent and treat infectious diseases, and improving diagnosis to control the spread of AMR. A direct effect of this activity was EU Regulation 2019/6 [42] on the prohibition of the use of antimicrobials in prophylaxis (directly and in feed) from 2022, limitation of the indications for use in metaphylaxis, indication and elimination of human drugs from animal production, obligation to monitor the sale and consumption of antimicrobials, and prohibition of the use of antibacterials as growth promoters in products from outside the EU. These restrictions are a very serious economic and social challenge but may promote the acceleration of efforts to develop and implement alternatives to the use of antibiotics. However, the impact of the reduced use of antibiotics on animal health and productivity is still debatable as it poses a challenge for veterinarians and breeders to seek alternatives or their combinations that will ensure similar animal husbandry parameters [27,32].

## 4. Factors Related to the Production and Implementation of Alternatives to Antimicrobials

Unfortunately, there are many factors that limit the rapid introduction of alternatives. In 2018, a workshop on strategic priorities for research of antibiotic alternatives in animal agriculture was held [43]. Experts analyzed priority research strategies for antibiotic alternatives by assessing critical points and economic determinants of the market success of products. As indicated by the experts, the most important ones include limited public and private sector funding for research on antibiotic alternatives. The insufficient interest in this type of research is associated with the expectation of obtaining confirmation of the positive effects of tested products that are already at an early stage of research [43]. Other factors contributing to the success of the product include economic availability for farmers and veterinary doctors, which is associated with higher production, handling, and distribution costs. A determinant of the success of a new product is also the volume of market demand, i.e., the use of the product only in a specific area (endemic for a given disease) or for a species-limited group of animals. The acceptance of the use of alternatives by farmers and veterinarians is an important part of the strategy of the introduction of new alternatives. In this case, veterinarians are the primary target group since they have the greatest influence on farmers’ awareness of animal health and principles of moderate use of new drugs [44,45]. The mechanism of action of such new alternatives should be clear, and their use (dosage, stability, and storage) should be easy and transparent for both the veterinarian and the farmer. Implementation of an alternative should not interfere with the production cycle and should not extend the treatment process (prolonging the time without milk production or suspension of the export of animals or products due to occurrence of infectious disease) [43].

The wider use of alternatives by veterinarians is usually associated with an analysis of long-term consequences for animal health and production economics. An analysis of the effects of the use of alternatives on the broad-sense animal health may be problematic. Depending on their mechanism of action, most alternatives either stimulate host immunity nonspecifically or regulate the gut microbiome. Most frequently, the effectiveness of such alternatives is difficult to predict in vivo, as most preliminary research is carried out in vitro or involves a genetically homogeneous group of animals [45]. The wide nontargeted action of alternatives, combined with the minimally differentiated specificity of the host organism (higher population-level variation), even within the same species, may result in substantially lower effectiveness of the substance used and even cause unpredictable side effects in young animals with an immature immune system [10]. Comparative evaluation of the effectiveness of in vitro and in vivo activity may pose another difficulty. Depending on the test, the effectiveness of the product can be measured as reduced mortality rates, a decline in the occurrence of clinical symptoms or their severity, and reduced occurrence of histopathological changes [46,47,48,49]. As a result, the evaluation of individual products may be ambiguous, and the results from different species or different age groups may be contradictory.

The introduction of any new alternative on the market is a complex process burdened with numerous economic and health risk factors, mainly faced by veterinarians and farmers. A particular problem is usually the lack of defined targeted action for most groups of alternatives compared to antibiotics, the limited in-vivo research on their efficacy, and the difficulty in predicting potential side effects in various groups of animals [36,44,50].

## 5. Alternatives and Their Applicability in Animal Production

The term “alternatives to clinical antimicrobials for animal production” comprises a vast range of available options, including products with different chemical structures and mechanisms of action, different target groups of animals, different routes of administration, and different expected effects [8]. Besides substances intended to replace antibiotics, there are also various management strategies related to the maintenance of biosecurity conditions, appropriate rearing conditions (e.g., reduction of animal density), and balanced diets [8,41]. Moreover, analysis of the occurrence of certain infectious diseases in a given area (endemic) or in a given country is conducted as a strategy for the reduction of antimicrobial resistance, and appropriate vaccination schedules and administrative activities related to the elimination of infectious diseases have been introduced [41,51] (Figure 1).

Cattle (beef and dairy industries), swine, and poultry are the primary target animal production groups and species [10,45]. There are many reviews presenting the use of alternatives in livestock production groups, depending on the animals’ age and species [8,52]. Some alternatives, e.g., probiotics, prebiotics, essential oils, organic acids, and in-feed enzymes, have been known and used for many years [8,53]. In turn, antimicrobial peptides, bacteriophages, molecular particles, predatory bacteria, and Cas9 and similar products with a targeted effect on specific parts of the bacterial-virulence-related genome still require further research, especially in terms of their use in animal production [54]. Their effectiveness depends on many factors, such as the route (food boluses, direct application into the udder, or injection), the time (the age of the animal), and the purpose of administration [8,45,55]. For an easier description of the alternatives, some authors divide these products into substances for growth promotion, disease prevention, and disease treatment. However, this division is quite conventional, as the same substances can often be used to achieve more than one goal [8,55]. As a rule, disease prevention substances are administered to healthy animals when the risk of disease increases or when some animals in the herd already show symptoms and it is necessary to control and stop the spread of infection. The similarities between substances used for disease prevention and those for growth promotion include administration to healthy animals and a longer duration of the treatment. Typically, such substances exert both effects since the activity as a growth promoter is partly related to the inhibition of growth or elimination of bacteria and an increase in body weight in healthy animals [8,10,37,55]. Each group of substances has its advantages and disadvantages and can be intended for the prevention or treatment of a specific group of diseases or can act more broadly. The most common nontargeted activity includes stimulation of the immune system nonspecifically or stabilization of the gastrointestinal microbiome [8,52,55].

Some alternatives are widely used commercially, although research on their effectiveness reveals opposite or divergent results. Other alternative substances, despite their proven effectiveness, are not used commercially for economic reasons, such as high production cost, low stability, or extreme specificity, allowing their use only in a limited group of recipients in endemic areas [8,10,55].

Since the alternatives for antimicrobial therapy constitute a huge and diverse group, it is very problematic to provide an exhaustive overview of all the substances available or those regarded as a potential alternative. Moreover, the issue to be considered is often not the application of only one alternative option but rather combinations of at least two different substances or even a combination of different action strategies and different groups of preparations. Therefore, we focused on only two groups of preparations with a targeted and usually selective effect on specific species or even strains of microorganisms, as is the case of antibiotics. Therefore, it is possible to predict the effectiveness of the alternatives more easily so that both bacteriophages and antibacterial peptides can be applied more widely in animal production in the future.

## 6. Bacteriophages

The history of the use of bacteriophages in the fight against bacteria dates back to the 1920s and 1930s, i.e., the preantibiotic era [56]. Initially, mainly Eastern European countries were interested; they conducted advanced research on the mechanism of action of bacteriophages, while Western European countries focused on discovering new therapeutic possibilities of classic antimicrobials [55,57]. Bacteriophages act like typical viruses with a very narrow target spectrum, switching the metabolism of the bacterial cell to the production of new particles released in the lytic cycle. The desired therapeutic effect of bacteriophages is the elimination of bacteria; however, the phage may enter the lysogenic cycle instead of the lytic cycle; thus, no therapeutic effect will be achieved [55]. The first commercial cocktail had already been developed in 1940 [10]. Currently, many commercial phage preparations, mainly cocktails (mixtures of several types of bacteriophages), are used in both human and veterinary medicine [58,59]. However, these preparations have both advantages and disadvantages. One of the greatest advantages is the well-known and targeted mechanism of phage action. Phages require specific receptors found only on the surface of bacterial cells, e.g., LPS or peptidoglycan, which are not present on the surface of host cells; therefore, they have no negative effects on human and animal cells [55,60], although some studies have confirmed the interaction between phages and eukaryotic cells [61,62]. Phages usually have a very narrow spectrum of activity, which is limited to one species (monovalent phage) or even to a specific pathotype [63]. However, polyvalent phages targeted to broad host ranges have been found as well [64,65]. The narrow spectrum, on the one hand, prevents negative effects on the entire host microbiome, as is the case for antibiotics [63]. On the other hand, this can be a significant limitation, especially in the treatment of polyetiological infections (syndromes) caused by several different species of bacteria [60]. The narrow spectrum of phages requires precise and quick determination of the etiological factor or factors of the infection before introducing targeted phage therapy. Hence, it may be a limitation in some cases [10]. Bacteriophages have the ability to multiply only at the site of infection, which is related to the lower frequency of administering phage preparations compared to antibiotics [63]. Bogovazowa et al. [66] have shown that phages can persist for one to several days after administration; however, their titer decreases drastically after 8–12 h [58,67]. The lifetime of phages depends on many factors, especially the route of supply. Depending on the type of infection, phages can be applied orally, topically, intraperitoneally, via intramuscular and intravascular injection, or as an aerosol [67]. When phages are administered orally, in order to reach the circulatory system, they must break down the intestinal barrier and avoid neutralization by the low pH of gastric acids. It has been confirmed that phages exhibit varied sensitivity to pH, digestive enzymes, and bile salts [58,68,69,70,71]; therefore, in the per os administration route, phages must be taken with neutralizing substances or must be microencapsulated in different carriers [67,68,69,70]. The half-life of phages in the host largely depends on the activity of the immune system [62]. Phage phagocytosis and antiphage immunoglobulin production are the main processes involved in the neutralization of phages. Phage inactivation time varies and depends on earlier contact with the phage and the development of immunological memory, but it generally reduces the number of active phages in the body [72,73]. Therefore, bacteriophage immunogenicity has also been described as one of the disadvantages and a cause of failure with repeated phage therapies due to rapid elimination from systemic circulation or inactivation through adaptive immune response [10].

However, no formation of antiphage immunoglobulins was observed in some patients, or their presence did not visibly affect phage activity [74], which in turn may correlate with the low titer of the initial dose of bacteriophages or with the short period of phage supply [62]. Phage therapy may not produce the desired effect if phages are introduced too late or in an insufficient dose [75]; therefore, the action of bacteriophages is limited in time. Selection of the optimal administration time and frequency and a sufficient dose is required before the beginning of therapy. It has been shown that a 16-h shift from the time of *E. coli* infection results in therapy failure [76]. Due to the large diversity of phage populations and target bacteria, the optimization of pharmacokinetic and pharmacodynamic parameters is not only a key element in the success of phage therapy but also one of its inconveniences [77]. Moreover, some authors have suggested a massive release of bacterial endotoxins during bacterial lysis, which may result in the development of a massive inflammatory cascade, leading to multiorgan damage as a side effect of phage therapy [10]. Nevertheless, studies conducted by Dufour et al. [78] have shown a higher concentration of released endotoxins during β-lactam therapy compared to phage therapy, confirming the greater safety of the latter.

Similar doubts were associated with the risk of transferring undesirable genetic elements [10]. According to the guidelines for the commercial production of phage preparations, there is no strictly established safe level of genetic variation for certain phage populations in subsequent cycles of propagation. However, similar to vaccine production, one of the tools of a quality control system is the use of metagenomics methods for each batch, allowing comprehensive monitoring of potential genetic variability [10,59]. Nevertheless, phages have to be subjected to a very restrictive quality assessment procedure, taking into account stability, identity, titer level, and possibility of contamination with other phages or bacterial proteins [59]. Such an extensive quality control system generates extra costs of production of commercial phage preparations [79]. Therefore, a commercial product may not fully meet the expectations of producers of food of animal origin due to the higher cost of phage therapy than that associated with the use of antibiotics. This probably limits the current market of commercial preparations for animals in comparison with human medicine [80,81].

The use of the so-called phage cocktails in poultry, piglets, and calves rather than single phage types is recommended since they support the treatment of polyetiological infections and reduce the development of resistance to such formulations [10,60].

In the case of poultry and pigs, phage therapy has been targeted at a specific group of pathogenic bacterial species, especially those with zoonotic potential. Recent EFSA reports [35] have revealed that the growing resistance of nontyphoidal *Salmonella*, *E. coli*, and *Campylobacter* spp. positively correlates with the number of new studies on potential phage therapy against these microorganisms [82,83,84,85]. In terms of the importance of phage therapy against *Salmonella* spp., mainly targeting such zoonotic serotypes as Enteritidis, Typhimurium, and Hadar [74,75,76,77], the main assumption is the preventive action will counteract colonization and reduce the excretion of these microorganisms into the environment through feces and prevent vertical infection in the case of poultry [83,86,87,88]. Colibacillosis occurs in all productive groups of animals and has many different forms, ranging from intestinal infections, respiratory syndromes, nervous system infections, and systemic infections (septicemia) to clinical or subclinical mastitis in cattle [85,89]. Depending on the form of infection, animal species and age, and bacteriophage titers used, promising results were obtained in the reduction of animal mortality [75,90,91] and limitation of the excretion of certain *E. coli* pathotypes [84]. A lethal effect was shown in vitro as well [85]. Phages have also been successfully used to reduce the number of *Campylobacter* bacteria in poultry [92,93]. However, bacteriophage-resistant *C. jejuni* populations have been reported [84,94].

Positive results of phage-therapy-induced reduction of bacterial counts have been obtained in the case of *Clostridium perfringens*, causing necrotic enteritis in poultry. Peptidoglycans, i.e., components of the bacterial cell wall, are their target site of action [79]. Moreover, it has been shown that endolysins released in the gastrointestinal tract exert a specific negative effect only on *C. perfringens* without killing other species of bacteria of this genus, which are part of natural gastrointestinal biota [95,96].

Both in vitro and in vivo studies have shown the effectiveness of bacteriophage-based therapy in the reduction of *Staphylococcus aureus* bacteria that causes mastitis in cattle [97,98]. However, in the case of pigs, Verstappen et al. [49] showed, in in-vitro and in-vivo assays, no inhibition of nasal colonization by MRSA strains with the use of specific phages.

The use of phage therapy in bees to combat *Paenibacillus larvae* infection, which causes American foulbrood (AFB), is also very promising [99]. *P. larvae* is a sporulating bacterium that persists in the environment due to its high resistance to unfavorable factors (including heat resistance), which makes it difficult to control. In addition, according to EU directives, hive-derived products, including honey, must not contain antibiotic residues [100].

Another use of the bactericidal action of phages is their direct application onto the surface of food of animal origin in the food production process. The use of phages is necessary in the case of food that cannot be subjected to inactivation of microorganisms by heat treatment or high pressure (changing the appearance or the nutrient content in some foodstuffs) [101]. Phage cocktails or single-phage types are used in the biocontrol of such bacteria as *Bacillus cereus*, *Campylobacter jejuni*, different serotypes of nontyphoidal *Salmonella*, *E. coli* (including the O:157 serotype), *Listeria monocytogenes, Staphylococcus aureus*, and even *Mycobacterium smegmatis* [102,103,104,105].

Although the effects of phage therapy in farm animals are quite promising, their wider use in animal production is still limited. Most research in this field is carried out in vitro; therefore, it is not possible to fully confirm the positive effects of therapy and exclude potentially negative effects that may arise through complex bacteriophage–bacterium–animal interaction [106]. Moreover, it is difficult to predict the effect of introducing phages on a massive scale in relation to various ecosystems, including natural ones [106]. In addition, there is still little connection between the scientific and biotechnology sectors related to the commercialization of research results that would allow the introduction of new preparations on the veterinary market [79,82,106].

## 7. Antimicrobial Peptides

Antimicrobial peptides (AMPs) are low-mass peptides with antibacterial, antiviral, antifungal, and even antiparasitic effects [107]. They constitute a vast and extremely diverse group of molecules (more than 3000 different AMPs are known at present), which are also produced by various taxa, including vertebrates, invertebrates, microorganisms, and plants [108,109]. Their action is mainly based on antimicrobial activity, but some of them also have anticarcinogenic effects and act as modulators of the immune system or repellants produced by plants [110]. The main antimicrobial mechanism of AMPs is based on incorporation and irreversible damage to the microbial cell membrane, resulting in cell lysis [107]. Some peptides can act synergistically by damaging the cell membrane and, thus, allowing the entry of other types of AMPs that have receptors inside the cell [111]. The mechanism of action of other peptides is based on the inhibition of DNA and protein synthesis or an effect on energy metabolism [112]. The most common effect of AMPs is the reduction, but not complete elimination, of the number of pathogenic microorganisms, which very often also depends on the dose administered [113].

Over 74% of all AMPs, constituting a very diverse group, are produced by animals. AMPs may be of bovine, caprine, ovine, porcine, chicken, turkey, and other animal species origin [114]. The expression of AMPs has been demonstrated in many tissues and cells (polymorphonuclear leukocytes and macrophages) [112]. Their properties are primarily suitable for the food and health industry, where they are used as food additives or for improvement of food storage [112,115,116,117]. Other AMPs have proven antibacterial properties against such animal pathogens as *Manheimia haemolytica*, *E. coli. Klebsiella pneumoniae*, *Pseudomonas aeruginosa,* other Gram-positive and Gram-negative bacteria, and fungi [118,119,120,121,122,123,124].

In vivo and in vitro studies confirmed that the use of lactoferrin against mastitis pathogens supported the treatment of infection in cows [125,126,127]. Wang et al. [128] showed that lactoferrin reduced the number of pathogens in suckling piglets as well. Additionally, the simultaneous use of cecropin, lactoferrin, and defensin in piglets was found to reduce the frequency of bacterial diarrhea in these animals [129].

AMPs are produced by both eukaryotes and prokaryotes, and those produced by bacteria are called bacteriocins [107]. Bacteriocins are characterized by a much greater specificity of action since their activity may be limited to only a few species of bacteria. They also have a much higher potential, as they are already active in pico- or nanomolar concentrations [108,130]. Unlike antibiotics, bacteriocins are ribosomally synthesized and may be inactivated by digestive enzymes in the gastrointestinal tract, although some of them may be resistant to low pH and proteases [130,131]. Bacteriocins are produced by both Gram-negative and Gram-positive bacteria. They are divided according to their chemical structure, mechanism of action, molecular weight, and spectrum [107,130]. Microcins and colicins, divided into two classes, are mainly produced by *E. coli* and other *Enterobacteriaceae* bacteria (*Shigella, Klebsiella, Yersinia*), while bacteriocins produced by Gram-positive bacteria (mainly lactic acid bacteria LAB) are more diversified and divided into four classes, depending on their molecular weight and thermostability [107]. Similar to bacteriophages, bacteriocins can be used for both combating microorganisms in animals and humans and as stabilizers to prevent spoilage and contamination of food [132]. To fight infections, purified bacteriocins are usually administered directly with food as feed additives or indirectly as bacteriocin-producing strains [8]. Therefore, it is quite difficult to clearly separate their use as antimicrobial peptides since many strains producing AMPs represent the probiotic group of microorganisms, which is another group of alternatives [133].

Pure bacteriocins are sensitive to pH and proteolytic enzymes, which limits their supply per os. If they have to be served via this route, only properly prepared boluses, pellets, or encapsulation forms must be used. Unfortunately, the increased costs of such therapy are a disadvantage [133].

The best-studied bacteriocin so far is nisin, which has been approved and recognized together with pediocin as a safe additive (GRAS status: generally regarded as safe) for food preservation [134] and accepted by the FDA as a food additive [135]. Its effectiveness against pathogens causing mastitis in cattle has also been confirmed in in-vitro and in-vivo studies [136,137]; additionally, its antibacterial activity against a substantially wider group of microorganisms, with the potential to be used in human medicine [138] and as a preservative in cosmetics, has been documented [139].

Research on the use of bacteriocins in animal production is mainly focused on specific groups of microorganisms that pose a health problem for poultry, pig, and cattle industries.

In the case of poultry, anaerobic bacteria responsible for necrotic enteritis (*C. perfringens*), avian pathogenic *E. coli* (APEC) causing colibacillosis, and zoonotic bacteria such as *Salmonella* spp. and *Campylobacter* spp. constitute the target groups of research on bacteriocin activity. Timbermont et al. [140] demonstrated the ability of perfrin, i.e., a bacteriocin expressing the NetB toxin that is produced by *C. perfringens*, to inhibit the growth of *C. perfringens* isolated from necrotic enteritis in poultry. In an in-vivo study, Wang et al. [141] confirmed the possibility of using microcin as a substitute for antibiotics in the poultry industry as this bacteriocin reduced the number of anaerobic bacteria and limited the frequency of *Salmonella* infections. Svetoch et al. [142] showed a reducing effect on *Campylobacter jejuni* in chicks that were infected experimentally with enterocin produced by *Bacillus polymyxa*. In an experimental infection of chickens with a selected strain of *E. coli*, Ogunbanwo et al. [143] showed a significant reduction in the clinical symptoms of colibacillosis after the supply of bacteriocins produced by a strain of *Lactobacillus plantarum*.

In pig production, the second most frequent cause of piglet deaths after enterotoxic *E. coli* (ETEC) is *Streptococcus suis*, which is responsible for a wide range of infections in this animal species [144]. Interestingly, as shown by Vaillancourt et al. [145], noninfectious strains of these bacteria, isolated from the tonsils of healthy pigs, are able to produce a bacteriocin with a lethal effect against pathogenic *S. suis* strains. The bactericidal activity of *S. suis* against a wider range of Gram-positive bacteria, i.e., streptococci and staphylococci, but not Gram-negative bacteria, was also confirmed by Sun et al. [115]. Mazurek-Popczyk et al. [146] also reported the interesting results of investigations of the antagonistic activity of *E. coli* strains isolated from healthy humans against zoonotic strains of the same species derived from pigs and cattle, which may be a promising alternative in the control of drug-resistant strains in animal production.

In cattle, the most common target group for the use of bacteriocins includes pathogens responsible for the infection of mammary glands [147]. Godoy-Santos et al. [148] showed an inhibitory effect of bovicin, a bacteriocin produced by *Streptococcus equinus*, against most Gram-positive isolates from mastitis but not against Gram-negative strains, including *E. coli*. The research conducted by Carson et al. [149] confirmed that bacteria that are the main cause of mastitis (non-*Aureus staphylococci*) may also be an important group of producers of bacteriocin that effectively inhibits not only animal-origin *S. aureus* but also human-origin methicillin-resistant *S. aureus* strains (MRSA). Lasagno et al. [147] showed the presence of genes encoding more than one bacteriocin in *Streptococcus uberis* strains isolated from mastitis; moreover, these strains showed inhibitory activity against other species of bacteria causing mastitis, especially coagulase-negative staphylococci.

Combined therapies, with the use of bacteriocins and bacteriophages or bacteriocins and other molecules, e.g., antibiotics, are used to increase the bactericidal effect of alternatives. Heo et al. [150] showed a synergistic inhibitory effect in vitro of a bacteriocin obtained from *Streptococcus hyointestinalis* and a cocktail of two lytic phages on the growth of *C. perfringens* isolated from the feces of poultry and pigs. LeBel et al. and Żbikowska et al. [96,151] showed a synergistic effect of combined nisin and β-lactams, as well as streptomycin and tetracycline, on *S. suis*, where a reduction of the MIC value for the antibiotics tested was achieved. Al Atya et al. [152] demonstrated a synergistic effect of the use of colistin, i.e., a LAB-produced enterocin, and nisin on not only biofilm-producing *E. coli* but also colistin-resistant strains.

As shown in these studies, the combination of different alternatives increases not only the bactericidal effect but also the synergism of action of AMPs and antibiotics through a reduction of the MIC value or even the virulence properties (e.g., the hemolytic activity of strains) [153,154,155].

However, as in the case of bacteriophages, the introduction of AMPs on a large scale into animal production requires confirmation of the effect in in-vivo conditions and in different target groups of animals. Unfortunately, most of the research on the antibacterial properties of AMPs is conducted in vitro [110]. Therefore, the results of studies that do not analyze the influence of conditions in a living organism may differ from those obtained in vivo [156].

Compared to antibiotics, the advantages of AMPs include less potent generation of resistance, for example, by weaker stimulation of the mutation frequency or a shorter time of killing bacteria, which prevents the appearance of resistant clones [157]. Another factor limiting the development of resistance to AMPs is the short persistence in the environment, which significantly reduces the possibility of selection pressure, as observed for antibiotics [158,159,160]. As in the case of bacteriophages, the greatest effectiveness is achieved when cocktails with well-known and properly selected compositions are used. Since small-molecule peptides are easy to reproduce, the technology of production is more convenient due to the easier process of synthesis of corresponding peptide variants [110,161]. Since it has been shown that some AMPs can only act against a narrow spectrum of species [162], the possibility of modification of their primary sequences allows the development of safe and stable AMPs that are specifically for the selected pathogens, with strictly targeted action against such bacteria as *E. coli, Salmonella* Pullorum, and *Pseudomonas aeruginosa* [160,163]. Certainly, the significant advantages of AMPs include their great diversity, which, in the future, may help to develop targeted therapy due to the possibility of designing AMPs with perfectly targeted actions. On the other hand, the low stability and sensitivity of AMPs to digestive enzymes and pH are usually described as limitations [106,160]. Therefore, the supply of pure peptides in the case of farm animals may be a weak point. The small number of reports on tests directly carried out on animals in natural conditions also makes it impossible to fully understand the mechanisms of action associated with the different results of in vitro and in vivo efficacy.

## 8. Conclusions

Despite the introduction of a large number of antiresistance strategies, including the marketing of alternatives to replace antibiotics, the actions taken consistently to accelerate the global downward trend in drug resistance does not appear to be entirely successful. Although developing countries have the largest share in the use of antibiotics (and further growth is expected until 2030), the production of food of animal origin in highly developed countries remains at the same level, and producers are still implementing antibiotic-dependent production systems. Meat consumption is constantly growing regardless of political systems and policies (24 kg/head in 1961, and as much as 43 kg/head in 2014) [4]. The use of antibiotics significantly lowers the economic risk of production as the maintenance and treatment costs decrease. In most countries, unlimited access to a cheap source of protein is still the main priority, rather than the increasing drug resistance, which is an abstract concept to the majority of the general public. An additional factor contributing to the failure of the global action counteracting drug resistance is the ineffective media awareness campaigns focused on the use of antibiotics [164,165]; hence, the public opinion does not exert effective pressure on the activity of state governments.

Although the 1970s and 1980s were the beginning of consumers turning towards safe and organic food, this was a drop in the ocean of global market expectations, likewise for the number of farms producing organic food. Restrictive requirements, longer production times, greater epidemiological risk, and higher production costs are additionally reduced by the willingness to convert most farms to this form of production [166,167].

Therefore, antibiotic alternatives are still a huge challenge for farmers, veterinarians, scientists, producers, and distributors. Targeted-action alternatives such as bacteriophages or AMPs seem to be most comparable to antibiotics in their effectiveness. Despite the very wide range of studies, mostly executed in vitro, both groups of alternatives have not obtained such a wide use status as antibiotics in animal production.

Thus, the accommodation of modern politics, the economy, and public health protection has become imperative, in which activities aimed at the development, implementation, and marketing of alternatives to antibiotics have a significant role to play. It is important to maintain the temporal predominance over microorganisms since they evolve much faster than it takes to develop and implement a new alternative for preventing and combating untreated infections.

## Figures and Tables

**Table 1:** International programs and organizations participating in global or local efforts to reduce drug resistance (data available in English).

Report/Project	Published by
A report on Swedish Antibiotic Sales and Resistance in Human Medicine (Swedres) and Swedish Veterinary Antibiotic Resistance Monitoring (Svarm)	Public Health Agency of Sweden and National Veterinary Institute Annual Report https://www.sva.se/en/our-topics/antibiotics/svarm-resistance-monitoring/swedres-svarm-reports/
Danish Integrated Antimicrobial Resistance Monitoring and Research Programme (DANMAP): Use of antimicrobial agents and occurrence of antimicrobial resistance in bacteria from food animals, food, and humans in Denmark	National Food Institute Statens Serum Institut (Denmark) Danish Ministry of Food, Agriculture and Fisheries and the Danish Ministry of Health in 1995. Annual Report https://www.danmap.org/
Swiss Antibiotic Resistance Report	Federal Office of Public Health FOPH (Switzerland) Annual Report https://www.bundespublikationen.admin.ch/cshop_mimes_bbl/
Annual Report	Scientific Board of ONERBA (the National Observatory of the Epidemiology of Bacterial Resistance to Antibiotics) France Annual Report http://onerba.org/onerba-in-english/
FINRES–Vet Finnish Veterinary Antimicrobial Resistance Monitoring and Consumption of Antimicrobial Agents	Finnish Food Authority (Finland) Annual Report https://www.ruokavirasto.fi/en/farmers/animal-husbandry/animal-medication/monitoring-of-antibiotic-resistance/finres-vet-reports/
UK Veterinary Antibiotic Resistance and Sales Surveillance Report	Veterinary Medicines Directorate (VMD; United Kingdom) Annual Report https://www.gov.uk/government/publications/veterinary-antimicrobial-resistance-and-sales-surveillance-2018
The European Union summary report on antimicrobial resistance in zoonotic and indicator bacteria from humans, animals, and food	European Food Safety Authority and European Centre for Disease Prevention and Control Based on Data Submitted by EU Member Countries Annual Report https://www.efsa.europa.eu/en/efsajournal/pub/6007
Sales of veterinary antimicrobial agents in 31 European countries in (given year) under the European Surveillance of Veterinary Antimicrobial Consumption project	the European Medicines Agency (EMA) Annual Report https://www.ema.europa.eu/en/veterinary-regulatory/overview/antimicrobial-resistance/european-surveillance-veterinary-antimicrobial-consumption-esvac
National Antimicrobial Resistance Monitoring System (NARMS) Annual Integrated Report	Food and Drug Administration (FDA) Annual Report https://www.fsis.usda.gov/wps/portal/fsis/topics/data-collection-and-reports/microbiology/antimicrobial-resistance/narms
OIE Annual Report on Antimicrobial Agents Intended for Use in Animals	World Health Organization for Animal Health Annual Report https://www.oie.int/scientific-expertise/veterinary-products/antimicrobials/
Global Action Plan On Antimicrobial Resistance	World Health Organization In 2015 http://www.emro.who.int/health-topics/drug-resistance/global-action-plan.html
The OIE Strategy on Antimicrobial Resistance and the Prudent Use of Antimicrobials	World Health Organization for Animal Health In 2016 https://www.oie.int/fileadmin/Home/eng/Media_Center/docs/pdf/PortailAMR/EN_OIE-AMRstrategy.pdf
EMA and EFSA Joint Scientific Opinion on measures to reduce the need to use antimicrobial agents in animal husbandry in the European Union, and the resulting impacts on food safety (RONAFA opinion)	EMA (European Medicines Agency) and EFSA (European Food Safety Authority) in 2017 https://www.efsa.europa.eu/en/efsajournal/pub/4666
Supporting Antimicrobial Stewardship In Veterinary Settings Goals For Fiscal Years 2019–2023	FDA (U.S. Food and Drug Administration) CENTER FOR VETERINARY MEDICINE In 2018 https://health.gov/healthypeople/tools-action/browse-evidence-based-resources/supporting-antimicrobial-stewardship-veterinary-settings-goals-fiscal-years-2019-2023-fda-center-veterinary-medicine
The 2019 WHO AWaRe classification of antibiotics for evaluation and monitoring of use	World Health Organization In 2019 https://www.who.int/medicines/news/2019/WHO_releases2019AWaRe_classification_antibiotics/en/
No Time To Wait: Securing The Future From Drug-Resistant Infections	World Health Organization Interagency Coordination Group (IACG) on Antimicrobial Resistance In 2019 https://www.who.int/antimicrobial-resistance/interagency-coordination-group/final-report/en/
Antibiotic Resistance Threats In The United States	Centers for Disease Control and Prevention In 2019 https://www.cdc.gov/drugresistance/pdf/threats-report/2019-ar-threats-report-508.pdf
